# Surface and Biocompatibility Outcomes of Chemical Decontamination in Peri-Implantitis Management

**DOI:** 10.3390/biomedicines13112748

**Published:** 2025-11-10

**Authors:** Alexandru Mester, Simion Bran, Marioara Moldovan, Ioan Petean, Lucian Barbu Tudoran, Codruta Sarosi, Andra Piciu, Dragos Ene

**Affiliations:** 1 Department of Oral Health, University of Medicine and Pharmacy “Iuliu Hatieganu”, 400012 Cluj-Napoca, Romania; 2 Department of Maxillofacial Surgery and Implantology, University of Medicine and Pharmacy “Iuliu Hatieganu”, 400012 Cluj-Napoca, Romania; 3 Institute of Chemistry “Raluca Ripan”, University Babes-Bolyai, 400294 Cluj-Napoca, Romania; 4 Faculty of Chemistry and Chemical Engineering, Babes-Bolyai University, 400084 Cluj-Napoca, Romania; 5 Faculty of Biology and Geology, Babes-Bolyai University, 400015 Cluj-Napoca, Romania; 6 National Institute for Research and Development of Isotopic and Molecular Technologies, 400293 Cluj-Napoca, Romania; 7 Department of Medical Oncology, University of Medicine and Pharmacy “Iuliu Hatieganu”, 400012 Cluj-Napoca, Romania; 8 Department of General Surgery, Faculty of Medicine, University of Medicine and Pharmacy Carol Davila Bucharest, 050474 Bucharest, Romania; 9 Bucharest Emergency University Hospital, 050098 Bucharest, Romania

**Keywords:** peri-implantitis, titanium implant, chemical decontamination, Ti6Al4V alloy, hydrogen peroxide, citric acid, EDTA, biocompatibility, osseointegration

## Abstract

**Background and Objectives:** Peri-implantitis is a biologically driven complication that jeopardizes dental implant longevity. While chemical decontamination is frequently employed as an adjunct to mechanical debridement, its impact on implant surface integrity and cellular compatibility remains insufficiently defined. This study aimed to evaluate the effects of several chemical agents used in peri-implantitis treatment on the surface morphology and potential biocompatibility of titanium dental implants. **Materials and Methods:** Twenty-five Ti6Al4V implants were exposed to one of the following agents: saline solution, 3% hydrogen peroxide, 40% citric acid, 17% EDTA, and a mixture (1:1) of citric (2%) and phosphoric (1N) acids. This in vitro study employed a 7-day immersion protocol to accentuate surface effects under controlled laboratory conditions, acknowledging that clinical exposures are substantially shorter. Surface topography was evaluated by Atomic Force Microscopy, while cellular response and corrosion products were assessed using Scanning Electron Microscopy. Surface roughness parameters were statistically analyzed. **Results:** Hydrogen peroxide induced selective corrosion of the β phase and formed a compact passivation layer that supported mesenchymal stem cell adhesion. Citric acid etched grain boundaries, producing localized roughness that also permitted cell proliferation. EDTA caused advanced grain dissolution and debris accumulation, increasing surface roughness but impairing cellular adhesion. The citric–phosphoric acid mixture led to the highest roughness values and visible corrosion debris. In all cases, macrostructural integrity of the implants was preserved. **Conclusions:** Chemical agents used in peri-implantitis treatment induce distinct surface alterations on titanium implants. Controlled use of hydrogen peroxide and citric acid may enhance surface biocompatibility, while aggressive protocols such as EDTA and acid combinations require caution due to their adverse effects on surface morphology and cellular response. These findings may inform the development of optimized decontamination protocols for clinical management of peri-implantitis.

## 1. Introduction

Peri-implantitis is a progressive inflammatory disease affecting the tissues surrounding dental implants, ultimately leading to bone loss and potential implant failure [1]. The condition is characterized by the destruction of peri-implant hard and soft tissues, often associated with bacterial infection and an exaggerated immune response. Reported prevalence rates vary widely (10% to 56%), reflecting differences in diagnostic criteria and types of patients [2,3]. Given its increasing incidence and the impact on implant longevity, peri-implantitis remains a major concern in periodontology and implant dentistry.

The treatment of peri-implantitis is particularly challenging due to the nature of biofilm formation on implant surfaces [4,5,6]. Unlike biofilms on natural teeth, which can be more easily disrupted, those forming on dental implants exhibit increased adherence and resistance to classical decontamination methods [4]. Various approaches have been proposed for managing peri-implantitis, including mechanical debridement, laser therapy, and adjunctive antimicrobial treatments [5,6]. However, none of these interventions have consistently achieved predictable, long-term clinical success [5,6].

Chemical decontamination agents have been investigated as adjuncts to mechanical debridement due to their ability to disrupt biofilms and reduce bacterial load [7,8]. Commonly used agents include chlorhexidine (CHX), hydrogen peroxide (H_2_O_2_), citric acid, and ethylenediaminetetraacetic acid (EDTA), each possessing distinct antimicrobial properties and mechanisms of action [7,8]. CHX is a widely used antiseptic known for its broad-spectrum antimicrobial effects, while H_2_O_2_ offers oxidative bacterial destruction [7,8,9]. Citric acid functions by altering the surface charge of bacterial cells, thereby reducing adhesion, and EDTA chelates divalent cations, destabilizing bacterial cell walls and implant biofilms [9]. Despite their potential benefits, concerns remain regarding their cytotoxicity and possible adverse effects on peri-implant tissues, emphasizing the need for further research [10] Conflicting reports exist regarding the impact of decontamination agents on titanium integrity and biocompatibility, with some studies suggesting beneficial passivation effects, while others report surface degradation or cytotoxicity [7,8,9,10].

In vitro studies play a crucial role in evaluating the effectiveness of chemical decontamination protocols under controlled conditions [10,11]. By simulating peri-implant environments, such studies help determine the ability of various agents to eradicate bacterial biofilms while preserving the integrity of implant surfaces and surrounding tissues [10,11].

Accordingly, this in vitro study aimed to evaluate the effects of commonly used chemical decontamination agents on titanium implant surfaces, focusing on surface morphology and preliminary biocompatibility rather than direct biofilm removal.

## 2. Materials and Methods

### 2.1. Experiment Protocol

This study did not require an ethics committee approval. A total of 25 titanium dental implants were used in this study. The implants were divided into five groups, each exposed to one of the following chemical agents: saline solution, 3% hydrogen peroxide (Vitalia Pharma, Ploiesti, Romania; pH 7.4), 40% citric acid (Cerkamed, Stalowa Wola, Poland), 17% ethylenediaminetetraacetic acid (EDTA) (Cerkamed, Stalowa Wola, Poland), and a mixture (1:1) of citric (2%,) and phosphoric (1N, Merk) acids. Implants were individually immersed in the corresponding chemical solutions for 7 days. Although a 7-day immersion period exceeds clinical contact durations, it was intentionally selected to magnify potential surface alterations for comparative purposes within an in vitro framework. At the end of the experiment, the implants were carefully removed from the solutions, rinsed with sterile saline to remove residual chemical agents, and dried for subsequent analysis. Each treatment group consisted of three independent titanium implant samples (*n* = 3) analyzed under identical experimental conditions.

### 2.2. Atomic Force Microscopy

Atomic Force Microscopy (AFM) investigation was effectuated on each titanium alloy screw’s surface, which was previously subjected to a corrosive environment. Topographical features were observed in tapping mode with a JSPM 4210 Scanning Probe Microscope (Jeol Co., Tokyo, Japan) using an NSC15-hard probe with a resonant frequency of 325 kHz and a force constant of 40 N/m. At least three different macroscopic sites at a scanned area of 5 × 5 µm^2^ were investigated, and the obtained images were further analyzed with specialized software WinSPM 2.0 (Jeol Co., Tokyo, Japan) measuring the surface roughness parameters Ra and Rq. For each independent implant, three distinct macroscopic areas (n = 3 per sample) were scanned, and mean roughness values were calculated to ensure measurement reproducibility. Roughness measurement and the benefit of revealing topographic features through the tapping mode were discussed in our previous studies [12,13].

### 2.3. Scanning Electron Microscopy

The Scanning Electron Microscopy (SEM) investigation was operated in high vacuum mode with a Hitachi SU8230 microscope (Hitachi Company, Tokyo, Japan). Samples were observed at an acceleration voltage of 30 kV. SEM observation was adjusted considering the aspects regarding stem cells observation described in our previous study [14], with the metallic body of the titanium screw ensuring a proper electrical conduction, and gold plating was necessary to view cell proliferation. SEM observations and cell adhesion evaluations were conducted on five independent implant samples per treatment group (*n* = 3).

### 2.4. Statistical Analysis

All quantitative data are presented as mean ± standard deviation (SD). Surface roughness parameters (Ra and Rq) measured by AFM, as well as cell adhesion frequencies derived from SEM morphological evaluation, were statistically analyzed to determine differences among the treatment groups. One-way analysis of variance (ANOVA) was employed to evaluate overall group differences. When statistically significant differences were detected (*p* < 0.05), Tukey’s post hoc test was conducted for pairwise multiple comparisons to identify specific intergroup differences. Statistical analyses were performed using dedicated software, with the level of significance set at *p* < 0.05.

## 3. Results

### 3.1. AFM Assessment

**Figure 1:**
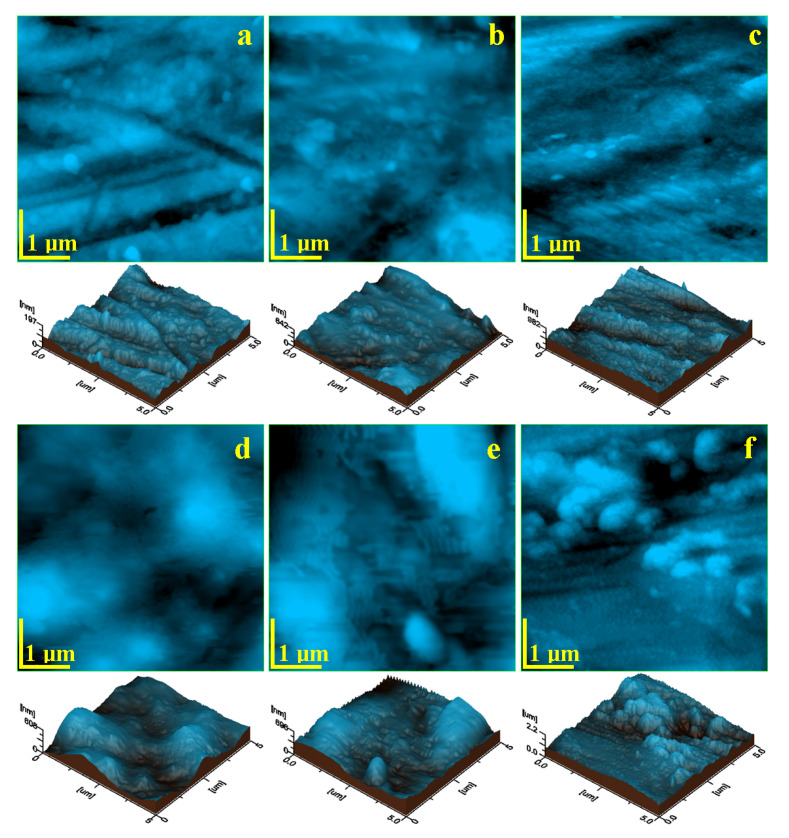
AFM topographic images of Ti6Al4V implants exposed to corrosive environments for 7 days: (**a**) control sample—unexposed, (**b**) saline solution, (**c**) hydrogen peroxide, (**d**) citric acid, (**e**) EDTA, and (**f**) mixture of citric and phosphoric acids.

The dental implant alloy’s microstructural features were clearly visible at this scale, revealing the relatively polyhedral α phase interconnected with the acicular β phase structure (Figure 1a). Portions of the polyhedral grains were observed with dimensions around 3–5 µm, forming larger structures of approximately 25 µm. The acicular β phase was more evident through the interconnected needles, measuring about 3–5 µm in length and 300–900 nm in diameter. The topographic surface appeared significantly uniform due to post-sintering calibration of the screws. After immersion in saline solution, Figure 1b shows good conservation of the α phase, while the β phase appears eroded, increasing surface asperities. Exposure to hydrogen peroxide resulted in significant erosion of the acicular β grains, while the α grains largely preserved their structure. A compact passivation layer formed of nanoparticles appeared over the scanned area (Figure 1c), preventing pitting. Citric acid exposure led to erosion of the α grains, which displayed rounded edges, and the upper layer of the β phase was locally depressed (Figure 1d). However, deeper pitting was not observed after 7 days of exposure. EDTA exposure (Figure 1e) showed advanced corrosion of the α phase and destructuring of the β phase, along with debris accumulation on grain surfaces, notably on α grains. Figure 1f demonstrates that phosphoric acid exposure led to breakdown of the citric acid-induced passivation layer and resulted in extensive deposition of sticky corrosion debris clusters across both α and β grains.

**Figure 2:**
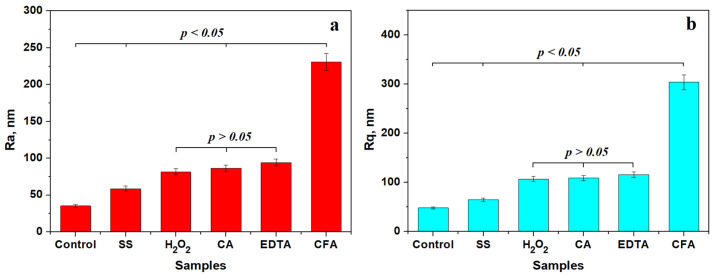
Mean values variation in surface roughness: (**a**) Ra and (**b**) Rq. SS: saline solution; H_2_O_2_: hydrogen peroxide; CA: citric acid; EDTA: ethylenediaminetetraacetic acid; CFA: mixture of citric and phosphoric acids. Plotted values are the mean of three independent measurements effectuated on different macroscopic areas on each investigated sample (*n* = 3).

Figure 2 presents the mean values of roughness variation plots with their statistical analyses. The control sample has the lower roughness value, forming the first relevant statistical group. Saline solution induced a mild increase in surface roughness; it was just enough to form the second statistical group characterized by mild corrosion of the β grains.

The third statistically relevant group comprises samples treated with hydrogen peroxide, citric acid, and EDTA, with *p* values greater than the significance level of 0.05. This statistical group is characterized by an increased corrosion of the outermost layers of β grains and slightly affecting the α grains border, followed by a subsequent passivation that prevented further development of the corrosion product.

The fourth relevant statistical group is formed by samples treated with a citric and phosphoric acids mixture. It is characterized by a strong increase in surface roughness associated with consistent erosion debris stacked over microstructural features.

### 3.2. SEM Assessment

**Figure 3:**
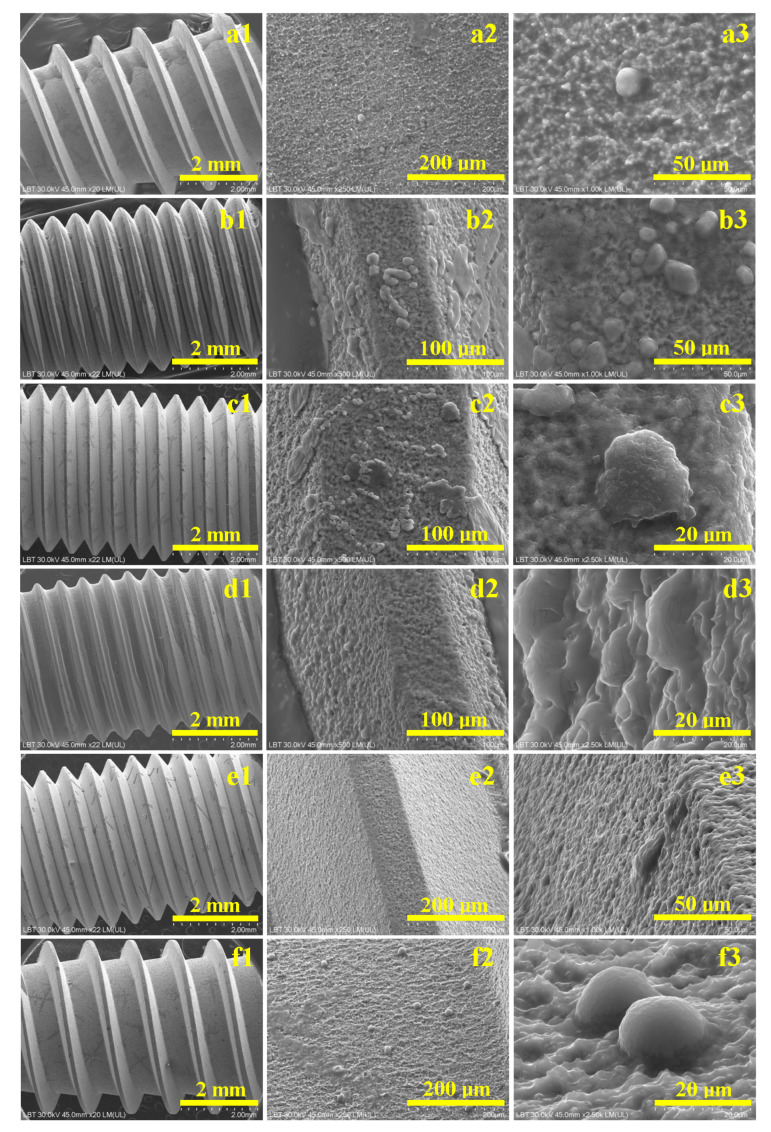
SEM images of Ti6Al4V screws exposed to corrosive environments for 7 days: (**a**) control sample—unexposed, (**b**) saline solution, (**c**) hydrogen peroxide, (**d**) citric acid, (**e**) EDTA, and (**f**) mixture of citric and phosphoric acids. (**1**) Macroscopic aspect, (**2**) microstructural detail, and (**3**) details on cell proliferation.

SEM images (Figure 3(a1)–(f1)) reveal that the overall macrostructure of the Ti6Al4V screws remained preserved after 7 days of exposure to corrosive environments. The thread helix showed no structural damage or obstructing deposits at the root across all samples.

In the control sample (Figure 3(a1)), as well as the sample treated with saline solution (Figure 3(b1)), significant cell clusters were observed on the thread crests. Phosphate solution residues, crystallized in dendritic forms, appeared in samples treated with hydrogen peroxide (Figure 3(c1)), EDTA (Figure 3(e1)), and the citric–phosphoric acid mixture (Figure 3(f1)). These features were not present on the sample treated with citric acid alone, suggesting reduced proliferation.

At higher magnification, the control sample displayed a flat, compact thread root composed of sintered particles (Figure 3(a2)). Mesenchymal stem cell nuclei, approximately 10 µm in diameter, were visible (Figure 3(a3)).

In the saline-treated sample, partial corrosion of β grains was visible, resulting in a roughened surface that supported cell attachment on thread crests and flanks (Figure 3(b2)). Rounded nuclei of 12–15 µm diameter were evident alongside rhombohedral phosphate crystals (Figure 3(b3)).

Hydrogen peroxide-treated samples showed preserved α grains and degraded β grains (Figure 3(c2)), producing roughened surfaces covered with cell clusters and buffer crystals. Figure 3(c3) highlights a 15 µm stem cell nucleus with visible protrusions.

Citric acid-treated samples exhibited pronounced surface roughness (Figure 3(d2)). Thread crests were devoid of cells, while the flanks hosted numerous cell clusters with nuclei between 10–15 µm (Figure 3(d3)).

EDTA-treated samples showed surface roughness due to corrosion debris accumulation (Figure 3(e2)). Phosphate crystals appeared at the crest–flank junction (Figure 3(e3)).

**Figure 4:**
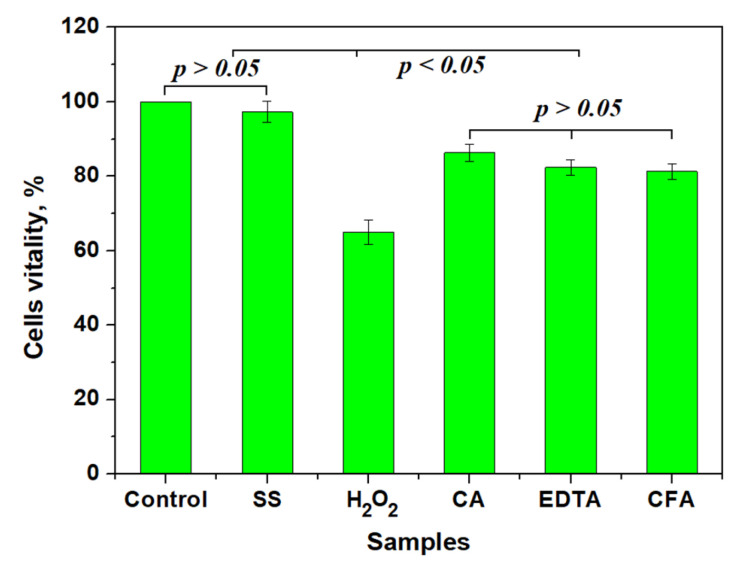
Mean values of relative cell adhesion on treated surfaces. SS: saline solution; H_2_O_2_: hydrogen peroxide; CA: citric acid; EDTA: ethylenediaminetetraacetic acid; CFA: mixture of citric and phosphoric acids. Three independent samples were investigated (n = 3).

The vitality of proliferated cells onto the eroded alloy surface was quantified. Thus, Figure 4 reveals a 100% vitality for the control sample, indicating the proper biocompatibility of the initial screws. Figure 4 shows that the corrosion effect influenced the cells’ vitality, forming three distinct statistical groups. The first group represents samples having vitality very close to the control, and includes samples exposed to saline solution. The second group contains only samples treated with hydrogen peroxide, having lower cell adhesion frequency. The third relevant statistical group is formed by samples treated with citric acid, EDTA, and the phosphoric acid mixture with citric acid. The cell adhesion frequency is about 83% for this group, indicating a good efficiency of cell proliferation, in good agreement with SEM observations.

Statistical analysis reveals significant differences within the groups (*p* < 0.05): it clearly indicates that saline solution more closely resembles the natural condition within patients’ bodies, and the acid treatment would be well supported by the adjacent tissue. Unfortunately, hydrogen peroxide antiseptic action is not selective in bacterial neutralization: it kills some of the tissue cells, and thus has a more invasive effect.

## 4. Discussion

The microstructure of the Ti6Al4V alloy, comprising polyhedral α grains and acicular β grains, plays a fundamental role in its response to corrosive environments and biological interfaces. The grain morphology observed in this study aligns with previous findings on powder metallurgy–processed titanium alloys, where α grains measure approximately 3–5 µm within larger domains (~25 µm), while the β phase consists of finer, needle-like structures [13,14]. Post-sintering calibration of the alloy contributes to reduced porosity and improved surface uniformity, providing a favorable baseline for surface characterization and cellular interactions [15,16].

Under exposure to saline solution, selective corrosion was evident. This behavior can be attributed to the electrochemical potential difference between the α and β phases. The α phase, predominantly titanium, passivates quickly through spontaneous oxide layer formation, while the vanadium-enriched β phase is more susceptible to chloride-induced degradation [17,18]. As a result, localized erosion and increased surface asperities were observed, consistent with β phase deterioration. The rougher surface morphology facilitated mesenchymal stem cell (MSC) attachment, particularly on thread crests and flanks, where both cells and phosphate crystals were detected, indicating a biologically active interface.

Hydrogen peroxide introduced oxidative stress through reactive oxygen species, leading to visible degradation of β grains while preserving α grains. A passivation layer formed across the alloy surface, composed of compact titanium oxides. This oxide film appeared to prevent deeper pitting, which is consistent with the literature describing peroxide-induced passivation at temperatures below 60 °C [19,20]. The resulting surface, characterized by nanoscale topography, provided an optimal environment for cell proliferation, with widespread MSC adhesion observed on passivated regions.

Citric acid, although a weak organic acid, exhibited significant corrosive action, particularly at the grain boundaries. The treatment induced rounding of α grains and shallow etching of β structures. Despite the absence of cell clusters on thread crests, MSC proliferation was detected on the flanks, within corrosion valleys. These results are consistent with earlier findings that citric acid can temporarily passivate the α phase while promoting β phase pitting under extended exposure [21,22,23]. Compared to parts manufactured by selective laser melting (SLM), which typically display higher porosity, the compactness of pressed powder metallurgy parts used in this study may have contributed to their greater resistance to acid corrosion [24].

EDTA, functioning as a chelating agent, had a pronounced corrosive effect by dissolving grain boundaries and forming loosely adherent corrosion debris. This debris increased surface roughness, but may have hindered individual cell adhesion. SEM images revealed phosphate crystal deposition, but no clear evidence of cellular attachment. The delayed passivation observed may be due to ongoing chelation at the surface, with surface stabilization requiring more time. This mechanism aligns with prior studies reporting EDTA’s ability to disrupt metal ion networks at the alloy surface [25,26].

Phosphoric acid, particularly when applied after citric acid treatment, disrupted any preformed passivation layers and produced a substantial amount of corrosion by-products. The sticky, particulate aggregates observed are consistent with synergistic effects described in the literature for combined organic and inorganic acid exposure [27,28].

Importantly, despite the different corrosion mechanisms induced by each chemical environment, the macrostructural integrity of all screw samples remained intact after 7 days of exposure. This structural preservation under corrosive and biological stress highlights the alloy’s robustness and supports its continued suitability for biomedical applications.

A clear correlation was observed between surface topography and biological response. Moderate roughness, generated by either partial corrosion (as seen with saline and hydrogen peroxide treatments) or surface passivation, promoted MSC adhesion. Because SEM cannot distinguish live from dead cells, these observations reflect relative adhesion and morphology rather than quantitative viability. In contrast, excessively rough or debris-laden surfaces (as observed with EDTA and the acid mixture) appeared less favorable for cell proliferation. This finding highlights the importance of controlled surface modification to optimize osseointegration and tissue compatibility.

Recent engineering-focused investigations have provided valuable insights into titanium alloy degradation and surface modification under corrosive conditions [29,30]. These studies emphasize that surface chemistry, microstructural phase distribution, and passivation dynamics critically influence corrosion resistance and subsequent biocompatibility, reinforcing the relevance of our surface-based evaluation. Although SEM and vitality assessments indicate trends in cellular adhesion, these results should be interpreted cautiously, as they represent limited in vitro indicators rather than comprehensive measures of biological compatibility [31,32].

These findings gain additional relevance when situated within the broader context of peri-implantitis management. Mechanical debridement using curettes, ultrasonic scalers, or air-abrasive devices serves as the cornerstone of therapy, but is often insufficient alone due to the complex implant geometry that protects bacterial biofilms from complete disruption [4,5].

Consequently, chlorhexidine (CHX) is widely employed for its broad-spectrum antimicrobial activity; however, systematic reviews and meta-analyses indicate its limited ability to fully eradicate biofilms on titanium surfaces, along with concerns about cytotoxicity and delayed healing [7,8]. Hydrogen peroxide offers oxidative antimicrobial action and may also enhance titanium surface passivation, aligning well with our observations of its moderate, cell-supportive surface alterations [21,22].

Citric acid is used clinically to remove biofilms and smear layers by altering local pH and chelating calcium, thereby promoting subsequent re-osseointegration. Our data confirm that while it effectively modifies the titanium surface to support MSC attachment within corrosion valleys, care must be taken to control exposure times to avoid excessive roughness [23,24]. EDTA similarly functions as a chelator that can remove endotoxins from titanium surfaces; however, our results—showing pronounced grain boundary corrosion and debris accumulation—suggest a need for cautious application, corroborating studies that indicate overexposure may lead to surface morphologies less favorable for early cellular attachment [27,28].

Recent meta-analyses emphasize the importance of combining these chemical methods with mechanical debridement. For example, a systematic review by Faggion and coworkers [5] noted modest improvements in probing depths and bleeding indices when adjunctive local chemotherapeutic agents were employed, although heterogeneity among study protocols prevents definitive conclusions. Likewise, Schwarz and coworkers [7] and Renvert and coworkers [4] demonstrated that while chemical agents can contribute to bacterial reduction, long-term evidence of consistent re-osseointegration remains limited.

Laser-assisted decontamination (using Er:YAG, diode, or CO_2_ systems) has also been studied extensively. However, meta-analyses conclude that while lasers may reduce bacterial loads and modestly improve clinical parameters, they do not consistently outperform chemical or mechanical methods in achieving stable peri-implant tissue outcomes [6].

A notable strength of this study is the evaluation of implant surface alterations using both AFM and SEM. This dual approach provided high-resolution insight into topographic changes as well as cellular responses, allowing a more thorough understanding of how different chemical agents influence both material integrity and biocompatibility. Another strength is the focus on clinically relevant decontamination agents. Hydrogen peroxide, citric acid, EDTA, and phosphoric acid are frequently employed in peri-implantitis management, yet their long-term impact on titanium implant surfaces remains insufficiently clarified.

However, several limitations should be acknowledged. First, the in vitro design, while valuable for isolating material and cellular interactions, does not fully replicate the complexity of the peri-implant environment, where biofilm composition, saliva, immune cells, and mechanical loading may alter the outcome of surface modifications. Additionally, the absence of biofilm models and the use of a limited cell adhesion assay constrained biological interpretation. Future work should include shorter, clinically relevant exposure times and biofilm-contaminated surfaces. Second, the seven-day exposure protocol, although useful for accentuating corrosion effects, did not reflect the shorter clinical contact times typical of chemical decontamination procedures. Third, this study employed a single implant alloy type and one surface morphology; variations in alloy processing, surface coatings, or implant design could yield different results. Finally, biological evaluation was restricted to mesenchymal stem cell adhesion and vitality, which, while informative, does not encompass the full spectrum of cellular and tissue responses relevant to osseointegration. Interpretations regarding passive-layer formation and β-phase etching are based solely on morphological evidence from AFM and SEM. Verification through complementary compositional or phase-identification techniques such as XPS, XRD, EDS/TEM, or EBSD will be pursued in future studies to substantiate these mechanistic insights.

Further studies should focus beyond in vitro conditions to in vivo models to confirm whether the observed surface alterations translate into improved osseointegration and clinical outcomes. Evaluating shorter, clinically relevant exposure times is essential to balance antimicrobial efficacy with preservation of surface integrity. In addition, testing other relevant cell types and exploring combined mechanical–chemical decontamination strategies may provide a more comprehensive understanding of how to optimize implant surface decontamination protocols.

## 5. Conclusions

Within the constraints of this in vitro study, titanium implants remained structurally stable after treatment with various chemical agents. These results emphasize controlled comparative analysis of surface alterations and biological responses, providing preliminary guidance for optimizing decontamination protocols. In contrast, EDTA and acid mixtures led to less favorable biological responses. These results highlight the potential of controlled surface modification to improve the biocompatibility of titanium implants. Further in vivo investigations and biofilm-based studies are necessary before extrapolating these findings to clinical peri-implantitis management.

## Data Availability

The original contributions presented in the study are included in the article, further inquiries can be directed to the corresponding author.
